# Supplementary material for: Genomic determinants of Furin cleavage in diverse European SARS-related bat coronaviruses
Source: Commun Biol. 2022 May 30;5:491. doi: 10.1038/s42003-022-03421-w (PMC9151638; doi:10.1038/s42003-022-03421-w)
Supplement: Supplementary file 2 — Supplementary Information [file 42003_2022_3421_MOESM2_ESM.pdf]

Supplementary Figures

Genomic determinants of Furin cleavage in diverse European SARS-related bat coronaviruses

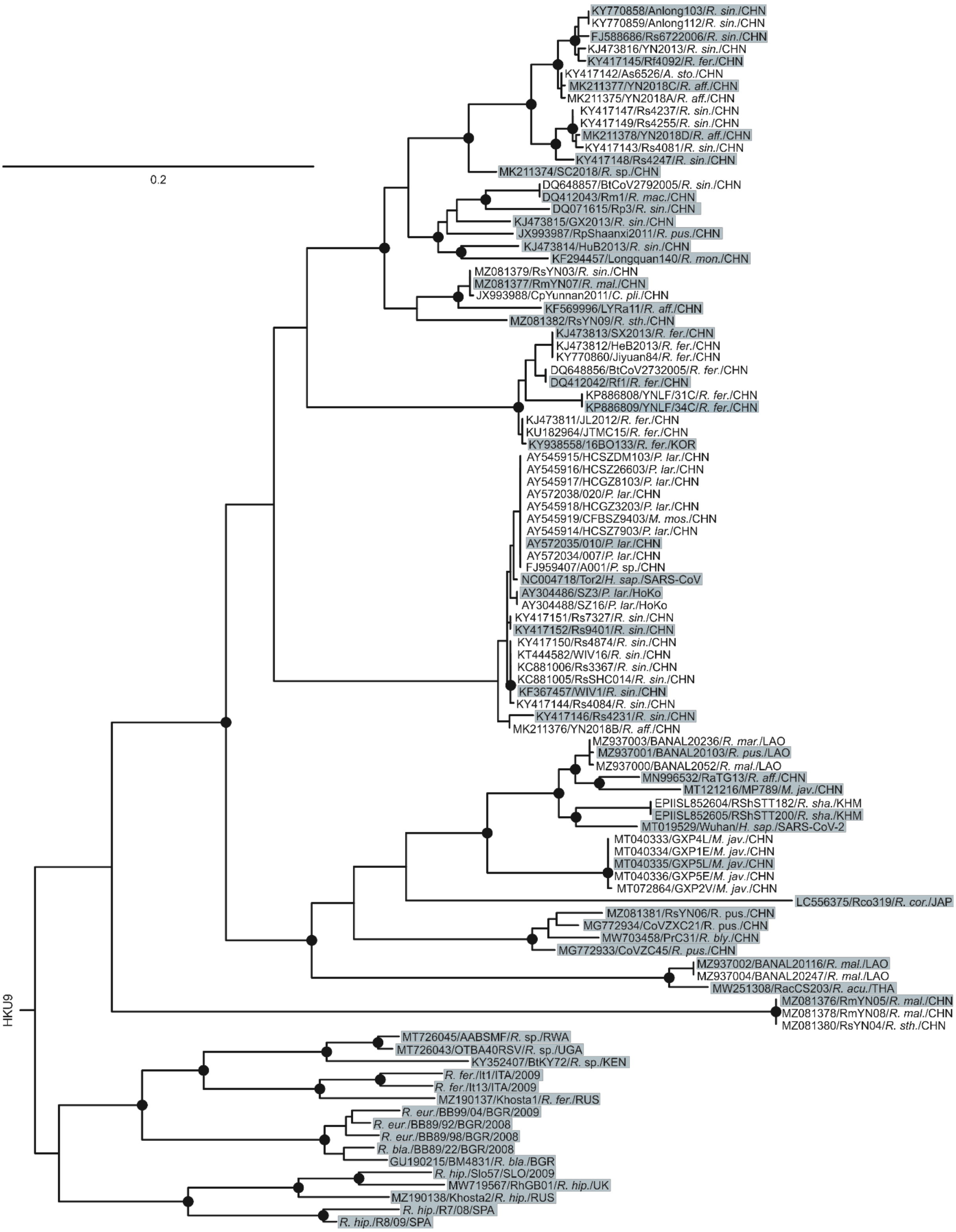

Supplementary Figure 1. Evolutionary relationships of SARS–related coronaviruses. Approximately-maximum-likelihood phylogeny showing the complete diversity of members of the

species *SARS-related Coronavirus* (*SrC*). The final dataset comprised 99 sequences (2 human, 12 civet, 6 pangolin and 79 bat-associated *SrC*). Sequences are named as followed: GenBank Acc. number/strain name/host species/country of detection. Circles at nodes indicate support of grouping in ≥ 90% of 1,000 bootstrap replicates. Scale bar represents nucleotide substitutions per site. Sequences highlighted in blue were chosen for phylogenetic analysis shown in Figure 1, representing the complete diversity of *SrC*. *A. sto., Asellicus stoliczkanus; C. pli., Chaerephon plicatus; H. sap*., *Homo sapiens*; *M. jav*., *Manis javanica*; *M. mos*, *Melogale moschata*; *P. lar*., *Paguma larvata*; *P.* sp.; *Paguma* species; *R. acu*., *Rhinolophus acuminatus*; *R. aff*., *Rhinolophus affinus*; *R. bla*., *Rhinolophus blasii*; *R. bly*., *Rhinolophus blythi*; *R. cor*., *Rhinolophus cornutus*; *R.Eur*., *RhinolophusEuryale*; *R. fer*., *Rhinolophus ferrumequinum*; *R. hip*., *Rhinolophus hipposideros*; *R. mac*., *Rhinolophus macrotis*; *R. mal*., *Rhinolophus malayanus*; *R. mar., Rhinolophus marshali*; *R. mon*., *Rhinolophus monoceros*; *R. pea*., *Rhinolophus pearsonii*; *R. pus*., *Rhinolophus pusillus*; *R. sha*., *Rhinolophus shameli; R. sin*., *Rhinolophus sinicus*; *R.* sp., *Rhinolophus* species*; R. sth*., *Rhinolophus stheno*; BGR, Bulgaria; CHN, China; ITA, Italy; JPN, Japan; KEN, Kenya; KHM, Cambodia; KOR, Korea; LAO, Laos; RUS, Russia; RWA, Ruanda; SLO, Slovenia; SPA, Spain; THA, Thailand; UGA, Uganda; UK, United Kingdom.

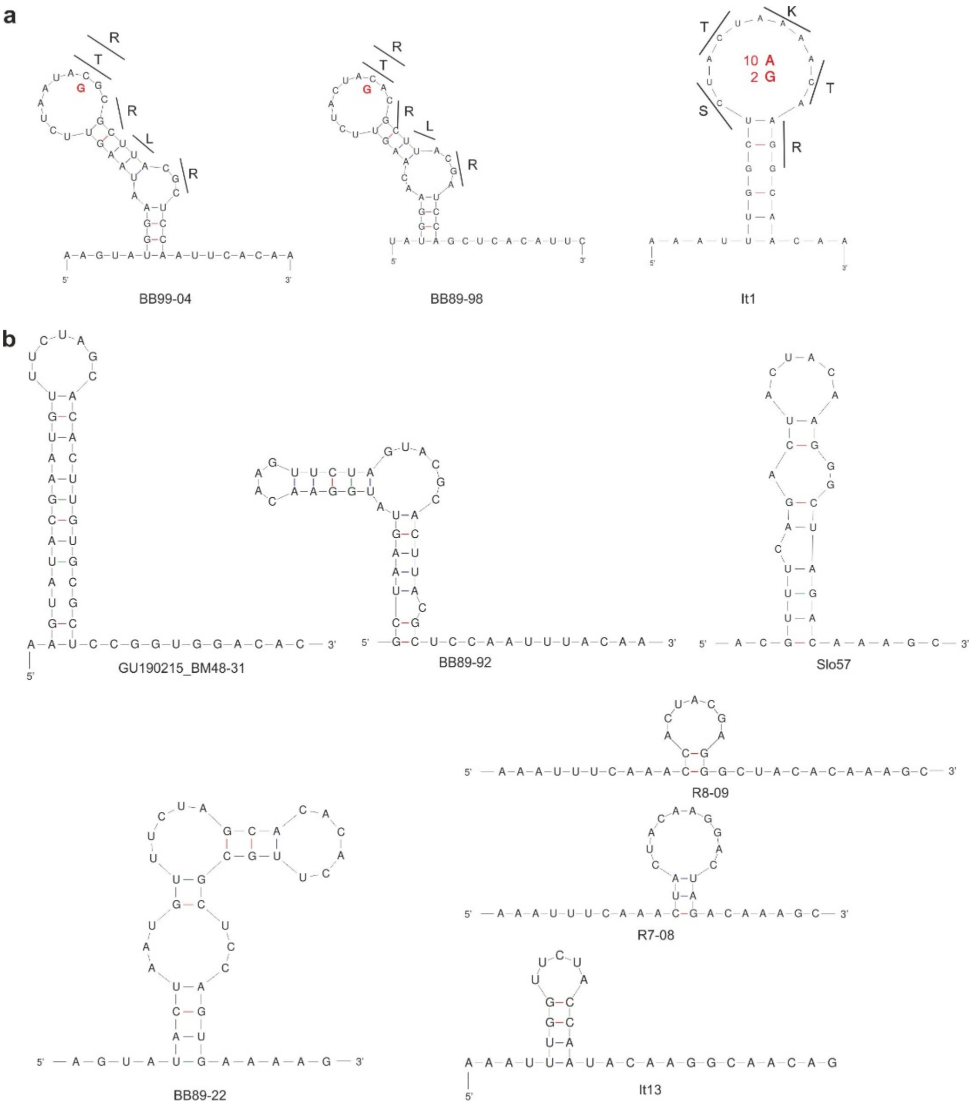

Supplementary Figure 2. Detailed predicted RNA secondary structures of the polybasic cleavage site of European bat SARS-related coronaviruses. a European bat SARS-related CoVs that may acquire FCS through nucleotide substitutions or insertions (shown in main text Figure 4b). Amino acids corresponding to codons forming the potential FCS are shown. Nucleotides and corresponding amino acids that change to a predicted FCS through nucleotide substitutions or insertions are marked in red. **b** European bat SARS-related CoVs that are not predicted to acquire FCS through nucleotide substitutions or insertions.

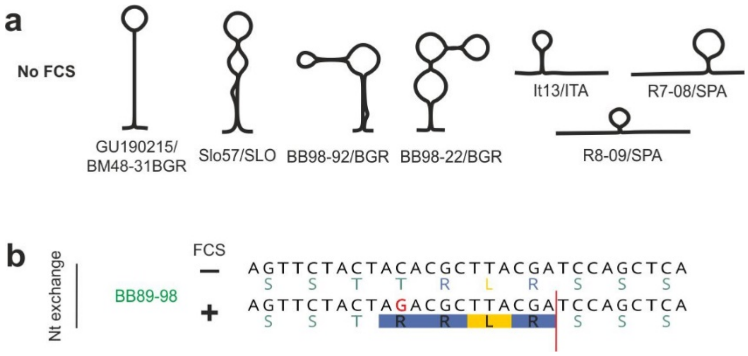

Supplementary Figure 3. a Predicted RNA secondary structures of the S1/S2 genomic regions of bat *SrC* that are not predicted to acquire an FCS through nucleotide substitutions or insertions. **b** Potential generation of FCS for bat *SrC* BB89-98 by nucleotide exchange. Sequence was trimmed in the figure for graphical reasons to highlight the FCS. The sequence used for FCS prediction was AKYGTSSTRRLRSSSHSIV; ProP score was 0.52.
