# Supplementary material for: Genomic determinants of Furin cleavage in diverse European SARS-related bat coronaviruses
Source: Commun Biol. 2022 May 30;5:491. doi: 10.1038/s42003-022-03421-w (PMC9151638; doi:10.1038/s42003-022-03421-w)
Supplement: Supplementary file 3 — Description of Additional Supplementary Files [file 42003_2022_3421_MOESM3_ESM.pdf]

Description of Additional Supplementary Files

File name: Supplementary Data 1
Description: Predicted furin cleavage sites with ProP scores for analysis in Figure 3.

File name: Supplementary Data 2
Description: RT-PCR primer sets for SrC spike glycoprotein gene characterization

File name: Supplementary Data 3
Description: Accession numbers of viruses included for analysis in Figure 3.
