# Supplementary material for: Genomic determinants of Furin cleavage in diverse European SARS-related bat coronaviruses
Source: Commun Biol. 2022 May 30;5:491. doi: 10.1038/s42003-022-03421-w (PMC9151638; doi:10.1038/s42003-022-03421-w)
Supplement: Supplementary file 7 — Reporting Summary [file 42003_2022_3421_MOESM7_ESM.pdf]

nature research

Corresponding author(s): Christian Drosten and Jan Felix Drexler

Last updated by author(s): 30.03.2022

Reporting Summary

Nature Research wishes to improve the reproducibility of the work that we publish. This form provides structure for consistency and transparency in reporting. For further information on Nature Research policies, see our Editorial Policies and the Editorial Policy Checklist.

Statistics

For all statistical analyses, confirm that the following items are present in the figure legend, table legend, main text, or Methods section.

n/a	Confirmed	
☒	☐	The exact sample size (*n*) for each experimental group/condition, given as a discrete number and unit of measurement
☒	☐	A statement on whether measurements were taken from distinct samples or whether the same sample was measured repeatedly
☒	☐	The statistical test(s) used AND whether they are one- or two-sided<br>*Only common tests should be described solely by name; describe more complex techniques in the Methods section.*
☒	☐	A description of all covariates tested
☒	☐	A description of any assumptions or corrections, such as tests of normality and adjustment for multiple comparisons
☒	☐	A full description of the statistical parameters including central tendency (e.g. means) or other basic estimates (e.g. regression coefficient) AND variation (e.g. standard deviation) or associated estimates of uncertainty (e.g. confidence intervals)
☒	☐	For null hypothesis testing, the test statistic (e.g. *F*, *t*, *r*) with confidence intervals, effect sizes, degrees of freedom and *P* value noted<br>*Give P values as exact values whenever suitable.*
☒	☐	For Bayesian analysis, information on the choice of priors and Markov chain Monte Carlo settings
☒	☐	For hierarchical and complex designs, identification of the appropriate level for tests and full reporting of outcomes
☒	☐	Estimates of effect sizes (e.g. Cohen's *d*, Pearson's *r*), indicating how they were calculated

Our web collection on statistics for biologists contains articles on many of the points above.

Software and code

Policy information about availability of computer code

Data collection	A tblastx search of Acc No. GU190215 was performed on the National Center for Biotechnology Information web server.<br>The NCBI Taxonomy website was used to download all sequences of the species Duvinacovirus, Merbecovirus, Setracovirus and Embecovirus.<br>A blast p search was of partial spike sequences of different coronaviruses was performed using the nr database on the National Center for Biotechnology Information web server.
Data analysis	UNAfold web server was used for modeling secondary structres of the spike cleavage site region.<br>ProP v.1.0b ProPeptide Cleavage Site Prediction was used for predicting furin cleavage sites.<br>Maximum-likelihood phylogenies were generated using FastTree Version 2.1.10 using a GTR substitution model and 1,000 bootstrap replicates.<br>A custom python script was used to extract GISAID sample IDs of sequences with complete or partial deletions of or within the SARS-CoV-2 FCS motif.

For manuscripts utilizing custom algorithms or software that are central to the research but not yet described in published literature, software must be made available to editors and reviewers. We strongly encourage code deposition in a community repository (e.g. GitHub). See the Nature Research guidelines for submitting code & software for further information.

Data

Policy information about availability of data

All manuscripts must include a data availability statement. This statement should provide the following information, where applicable:

- Accession codes, unique identifiers, or web links for publicly available datasets
- A list of figures that have associated raw data
- A description of any restrictions on data availability

All nucleotide sequences generated within this study were submitted to GenBank under accession numbers KC633198, KC633201, KC633203, KC633204, KC633205, KC633209, KC633212, KC633217 and KR559017. KR559017 represents the full spike gene of the Bulgarian SrC BB99-04.
MiSeq sequence reads have been deposited in the European Nucleotide Archive (ENA) under the study accession code PRJEB51900 with the read file accession codes ERR9434412 and ERR9434413.

Field-specific reporting

Please select the one below that is the best fit for your research. If you are not sure, read the appropriate sections before making your selection.

☒ Life sciences ☐ Behavioural & social sciences ☐ Ecological, evolutionary & environmental sciences

For a reference copy of the document with all sections, see nature.com/documents/nr-reporting-summary-flat.pdf

Life sciences study design

All studies must disclose on these points even when the disclosure is negative.

Sample size	Not applicable
Data exclusions	No data were excluded from the study.
Replication	Not applicable
Randomization	Not applicable
Blinding	Not applicable

Reporting for specific materials, systems and methods

We require information from authors about some types of materials, experimental systems and methods used in many studies. Here, indicate whether each material, system or method listed is relevant to your study. If you are not sure if a list item applies to your research, read the appropriate section before selecting a response.

Materials & experimental systems

n/a	Involved in the study	
☒	☐	Antibodies
☒	☐	Eukaryotic cell lines
☒	☐	Palaeontology and archaeology
☐	☒	Animals and other organisms
☒	☐	Human research participants
☒	☐	Clinical data
☒	☐	Dual use research of concern

Methods

n/a	Involved in the study	
☒	☐	ChIP-seq
☒	☐	Flow cytometry
☒	☐	MRI-based neuroimaging

Animals and other organisms

Policy information about studies involving animals; ARRIVE guidelines recommended for reporting animal research

Laboratory animals	This study did not involve any laboratory animals.
Wild animals	R. euryale, R. blasii, R. ferrumequinum and R. hipposideros were sampled in four countries (Bulgaria, Italy, Slovenia, Spain). All animals were handled according to national and European legislation for the protection of animals (EU council directive 86/609/EEC). No animals were sacrificed during this study. All animal handling and sampling was done by trained personnel, with animal safety and comfort as the first priority during minimally invasive sampling (collection of faeces). Captured bats were freed from nets immediately and put into cotton bags for 2 to 15 min to allow them to calm down before examination. While being kept in bags, bats produced fecal pellets that were transferred to 500µl RNAlater RNA stabilization solution (Qiagen, Hilden, Germany) for sample

	processing.
Field-collected samples	The study did not involve samples collected from the field.
Ethics oversight	Licenses for sampling of bats using mist nets, hand nets or harp traps were obtained from the respective countries and authorities: Bulgarian Ministry of Environment and Water, permit No. 192/26.03.200913, Italien Ministry of the Environment, permit No. 192/26.03.200952, Slovenian Environment Agency, permit No. 35701-80/200453, Service for the Biodiversity Conservation of the Rural Counseling of the Xunta de Galicia, Spain, permit No. 52/2010 n.s. 13697.

Note that full information on the approval of the study protocol must also be provided in the manuscript.
